# IL-8 Induces Neutrophil Extracellular Trap Formation in Severe Thermal Injury

**DOI:** 10.3390/ijms25137216

**Published:** 2024-06-29

**Authors:** Ali Asiri, Jon Hazeldine, Naiem Moiemen, Paul Harrison

**Affiliations:** 1 Institute of Inflammation and Ageing, University of Birmingham, Birmingham B15 2TT, UK; ama911@student.bham.ac.uk (A.A.); j.hazeldine@bham.ac.uk (J.H.); naiem.moiemen2@nhs.net (N.M.); 2 The Scar Free Foundation Centre for Conflict Wound Research, Queen Elizabeth Hospital Birmingham, Birmingham B15 2GW, UK; 3 NIHR Surgical Reconstruction and Microbiology Research Centre, University Hospitals Birmingham Foundation Trust, Mindelsohn Way, Birmingham B15 2WB, UK

**Keywords:** burns, sepsis, neutrophil extracellular traps (NETs), interleukin-8 (IL-8), cell-free DNA (cfDNA), DNase, actin

## Abstract

Neutrophil extracellular traps (NETs) have a dual role in the innate immune response to thermal injuries. NETs provide an early line of defence against infection. However, excessive NETosis can mediate the pathogenesis of immunothrombosis, disseminated intravascular coagulation (DIC) and multiple organ failure (MOF) in sepsis. Recent studies suggest that high interleukin-8 (IL-8) levels in intensive care unit (ICU) patients significantly contribute to excessive NET generation. This study aimed to determine whether IL-8 also mediates NET generation in patients with severe thermal injuries. IL-8 levels were measured in serum samples from thermally injured patients with ≥15% of the total body surface area (TBSA) and healthy controls (HC). Ex vivo NET generation was also investigated by treating isolated neutrophils with serum from thermal injured patients or normal serum with and without IL-8 and anti-IL-8 antibodies. IL-8 levels were significantly increased compared to HC on days 3 and 5 (*p* < 0.05) following thermal injury. IL-8 levels were also significantly increased at day 5 in septic versus non-septic patients (*p* < 0.001). IL-8 levels were also increased in patients who developed sepsis compared to HC at days 3, 5 and 7 (*p* < 0.001), day 10 (*p* < 0.05) and days 12 and 14 (*p* < 0.01). Serum containing either low, medium or high levels of IL-8 was shown to induce ex vivo NETosis in an IL-8-dependent manner. Furthermore, the inhibition of DNase activity in serum increased the NET-inducing activity of IL-8 in vitro by preventing NET degradation. IL-8 is a major contributor to NET formation in severe thermal injury and is increased in patients who develop sepsis. We confirmed that DNase is an important regulator of NET degradation but also a potential confounder within assays that measure serum-induced ex vivo NETosis.

## 1. Introduction

Responsible for around 180,000 deaths annually, burns are a global significant public health concern [1]. Thermal injuries result from tissue damage due to exposure to heat (e.g., fire, hot liquids), chemicals (e.g., acids, alkalis) or electricity (e.g., lightening, power lines) and present significant clinical challenges because of their varied causes and severity [2].

Major thermal injuries lead to a systemic inflammatory response syndrome (SIRS) characterised by an elevation in circulating pro-inflammatory cytokines [3]. SIRS is accompanied by a compensatory anti-inflammatory response syndrome (CARS) that is characterised by immune system impairment, which results in profound immunoparesis and an increased risk of subsequent infection and sepsis [4].

Neutrophils are the predominant type of human-circulated leukocyte and play a crucial role in the innate immune system, providing an immediate front-line defence against pathogens such as bacteria, yeast, and fungi [5]. Neutrophils can eliminate pathogens through three distinct mechanisms: phagocytosis and the production of reactive oxygen species (ROS), degranulation, and the formation of neutrophil extracellular traps (NETs). Phagocytosis involves the engulfment and subsequent killing of bacteria. Degranulation refers to the release of such antimicrobial proteins as neutrophil elastase and myeloperoxidase, which aid in destroying pathogens. The process of neutrophils forming NETs was initially described by Brinkmann et al. in 2004 [6]. NETs are web-like structures comprised of chromatin, histones and antimicrobial proteins released by neutrophils to capture and neutralise pathogens [7].

The mechanisms behind NET formation are still not fully understood and they occur not only through diverse stimulations and multiple signalling pathways, but with unknown interdependence. Although the major pathways described include suicidal and vital NETosis or mitochondrial DNA (mtDNA) release [8,9], it remains controversial how both the suicidal and more rapid vital NETosis pathways co-exist and which are more relevant in vivo. In the originally described suicidal pathway induced by phorbol 12-myristate 13-acetate (PMA) or bacteria, neutrophils release extracellular chromatin via the NADPH oxidase 2 (NOX-2) dependent pathway, resulting in cell death [10,11]. NOX-2 is an enzyme complex responsible for generating ROS within the neutrophil and promotes chromatin de-condensation and breakdown of nuclear and granular membranes [7,10]. The vital NETosis (NADPH oxidase independent) pathway can be induced through stimuli such as lipopolysaccharide (LPS) from Gram-negative bacteria and lipoteichoic acid (LTA) from Gram-positive bacteria, interleukin-8 (IL-8) and tumour necrosis factor-alpha (TNF-α). These induce cytoskeletal rearrangements within the neutrophil, leading to the formation of vesicles containing chromatin and antimicrobial proteins [12,13]. The mtDNA release pathway is initiated by neutrophils in response to infection or stress within the mitochondria. MtDNA, along with nuclear chromatin and other components of the NET, is subsequently transported into the cytoplasm and released extracellularly [14]. Despite the advances in this field, more research is required to fully understand the regulation and mechanisms of NETosis.

Although NETs are widely recognised as an initial line of defence against infection, excessive NETs are double-edged swords as they have also been observed in immunothrombosis [15]. This promotes disseminated intravascular coagulation (DIC) [16,17], impairing microcirculation flow and contributing to multiple organ failure (MOF) [18]. Deoxyribonuclease I (DNase I) is important for the homeostatic regulation of NETs [19]. Dinsdale et al. (2020) and Hazeldine et al. (2021) demonstrated that DNase activity in serum is not only capable of eliminating NETs in vitro, but it also can be inhibited by tissue-derived actin in burns and trauma resulting in excessive unregulated NET formation [20,21].

Excessive NETosis can therefore occur during acute inflammation and sepsis. Laggner et al. (2022) and Otawara et al. (2018) have illustrated that severe thermal injuries are associated with excessive NET production in humans and rodents, respectively [22,23]. However, NET formation is not just initiated directly by microbes and PAMPs, but can also be triggered by a range of pro-inflammatory mediators, including TNF-α, IL-8, activated platelets and activated endothelial cells [24]. Furthermore, platelet–neutrophil interactions have been shown to substantially contribute to initiating thrombosis via the rapid generation of NETs during vital NETosis [25]. Although we and others have demonstrated that NETs are generated in patients with severe thermal injury, the pathophysiological mechanism(s) of NET generation remains unclear.

IL-8 is a key cytokine and inflammatory mediator, which induces adhesion molecule expression on the vessel wall, stimulating neutrophil rolling, adhesion and migration to the tissues [26,27]. The biological effects of IL-8 are mediated by its binding to CXCR1 and CXCR2, which are cognate G-protein-coupled CXC chemokine receptors. This binding initiates a phosphorylation cascade that stimulates chemotaxis and neutrophil activation as components of the inflammatory response [28,29,30]. However, dysregulated signalling along the IL-8/CXCR1/2 axis has been recognised as a potential contributor to immunopathology, which results in prothrombotic activation, neutrophil degranulation and NET generation [31,32]. Teijeira et al. (2021) have illustrated that the concentrations of IL-8 needed to trigger NETosis in human neutrophils are at least double those necessary for chemoattraction. IL-8-induced NETosis relies upon the activation of different pathways compared to chemotaxis, with NET formation less reliant on G-proteins and more dependent upon ROS generation [33]. Abrams et al. (2019) demonstrated that high IL-8 levels in plasma or serum obtained from intensive care unit (ICU) patients significantly contribute to NET formation [34]. In addition, Nie et al. (2019) have demonstrated that IL-8 contributes to excessive NETosis in diffuse large B-cell lymphoma patients [35]. According to Kilic et al. (2023), COVID-19 patients showed increased IL-8 levels, and serum from these patients induced NETosis in vitro, which was reduced by co-culture with the IL-8 inhibitor, reparixin. [36]. Here, based on these observations, we aimed to determine the potential role of IL-8 in NET generation in patients with severe thermal injury.

## 2. Results

### 2.1. Circulating IL-8 Levels in Thermal Injury

**Figure 1:**
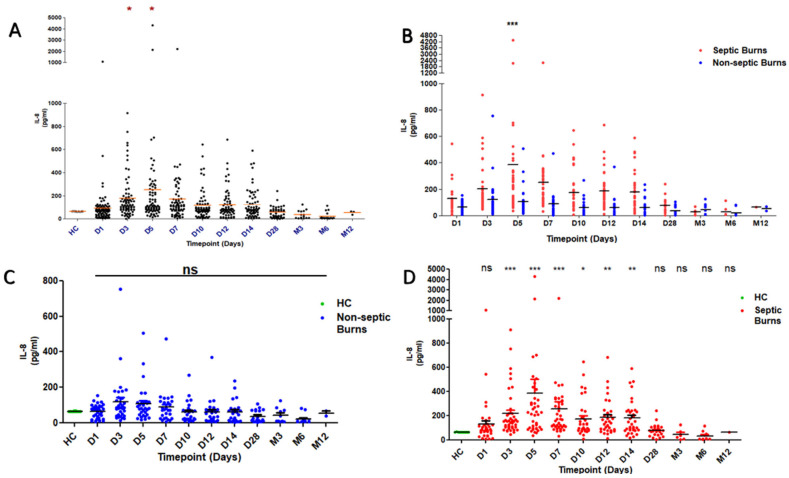
**IL-8 levels in thermal injury:** (**A**) Shows IL-8 levels in Healthy Control (HC) and burns serum samples from admission to D14, D28, M3, M6, and M12. IL-8 levels were significantly increased on days 3 and 5 compared to HC. (**B**) A comparison between IL-8 levels in burns patients who developed sepsis or not. IL-8 levels were significantly higher in septic than non-septic burns on D5. (**C**,**D**) illustrate the IL-8 level differences for non-septic and septic burns, respectively, compared to HC IL-8 levels. Non-septic burns were not significantly different from HC. In burns patients who developed sepsis, IL-8 levels were significantly increased on days 5–14 compared to HC. *p* value *** < 0.001, ** < 0.01, * < 0.05. (ns) not significant.

Serum IL-8 levels were measured in healthy controls (HC = 10) and burn patients from the day of hospital admission to month 24 post-burn. Figure 1A illustrates longitudinal IL-8 levels in burns patients and HC, with IL-8 levels significantly higher at days 3 and 5 post-burn (*p* < 0.05). Figure 1B shows the comparison of IL-8 levels between burns patients who did or did not develop sepsis. IL-8 concentrations were significantly higher at day 5 post-burn in patients with sepsis (*p* < 0.001). A comparison of IL-8 levels between patients with and without sepsis and HC found IL-8 was significantly increased in patients who developed sepsis compared to HC at days 3, 5, 7 (*p* < 0.001), 10 (*p* < 0.05) 12 and 14 (*p* < 0.01) (Figure 1C,D).

### 2.2. NET Formation Can Be Induced by IL-8 in Serum from Severe Burns Patients

**Figure 2:**
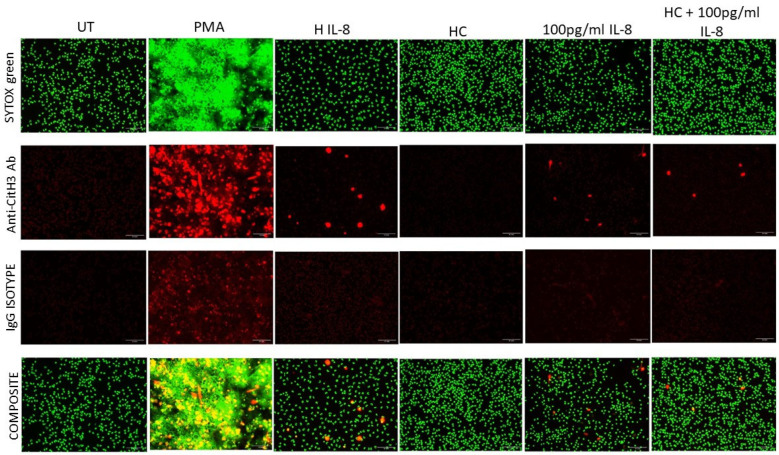
**IL-8 induces ex-vivo NETosis**. Isolated neutrophils were untreated as a negative control (UT) or stimulated with positive control (PMA), burn patient serum containing a high IL-8 (H IL-8) level (725.49 pg/mL), HC serum, 100 pg/mL recombinant IL-8 or HC serum supplemented with 100 pg/mL IL-8 for 4 h. Induced NETs were labelled with SYTOX Green and anti-citH3 Ab. Composite between cells double labelled with SYTOX and anti-citH3 Ab shows co-localisation. The scale bar represents 0.1 mm. Images are representative of five independent experiments.

Fluorescence microscopy was used to assess the capacity of burns serum containing IL-8 to induce ex vivo NET generation by neutrophils isolated from HC. Figure 2 shows a comparison of NET formation by untreated and PMA-treated neutrophils, which served as negative and positive controls, respectively. Untreated neutrophils retained their normal nuclear morphology and size. PMA stimulation induced NETs consisting of extracellular chromatin labelled with SYTOX Green and an anti-CitH3 Ab. Neutrophils treated with serum containing high levels of IL-8 generated NETs. In contrast, neutrophils stimulated by serum from HC did not generate NETs (Figure 2). Treatment with 100 pg/mL of recombinant IL-8 in either RPMI medium or HC serum-induced NETs (Figure 2).

**Figure 3:**
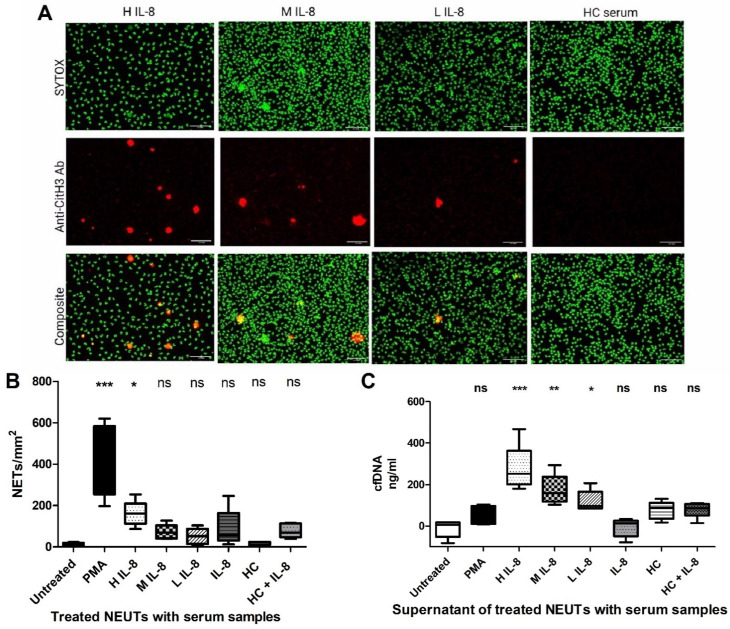
**NET formation correlates with IL-8 levels in burns serum:** (**A**) Fluorescent microscopy of NETs induced by burns serum containing either high (H), medium (M) or low (L) levels of IL-8, compared to HC serum. Generated NETs were labelled with SYTOX green and an anti-CitH3 Ab. (**B**) Quantification of NETs in all treatment conditions. (**C**) CfDNA levels in the supernatants of treated neutrophils. [ANOVA *p* value] *** < 0.001, ** < 0.01, * < 0.05. (ns) not significant. Data are representative of n = 5.

We next determined whether there was a dose–response effect of serum IL-8 levels on NET generation. Serum samples were classified into three groups: high IL-8 serum (H IL-8) contained very high levels (>500 pg/mL); medium IL-8 serum (M IL-8) contained between 150 and 500 pg/mL and low IL-8 (L IL-8) contained <150 pg/mL. Figure 3A illustrates a clear dose–response effect of serum IL-8 with H IL-8 serum increasing NET formation > M IL-8 and L IL-8 serum. Quantification of generated NETs using ImageJ software (Figure 3B) confirmed that H IL-8 serum and the PMA positive control significantly increased NETosis compared to untreated control (*p* < 0.05). Measurement of supernatant cell-free DNA (cfDNA) levels (Figure 3C) also confirmed a dose–response effect of serum IL-8 levels on NET generation. Given that DNase activity is present within serum, the data would suggest that cfDNA is being released from NETs in the presence of serum, as demonstrated previously by Dinsdale et al. (2020) [20].

### 2.3. Inhibition of DNase Increases NET Generation by IL-8 Serum

**Figure 4:**
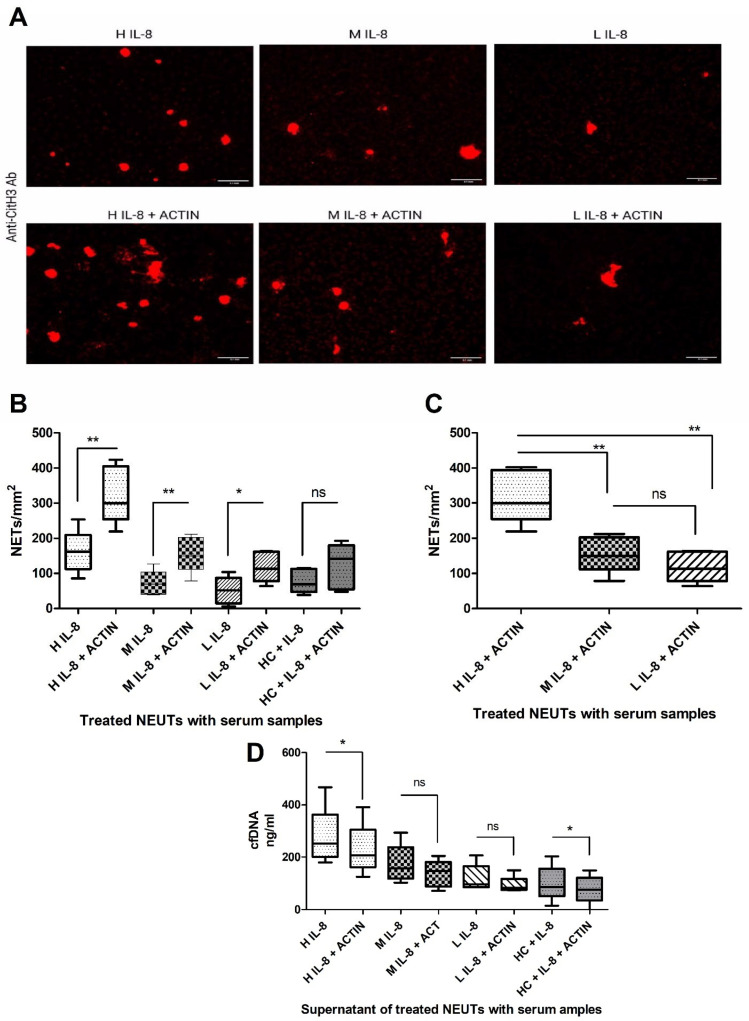
**DNase I inhibition induces more extensive NET formation by serum IL-8:** (**A**) Fluorescent microscopy of neutrophils stimulated with high (H), medium (M) or low (L) IL-8 levels in the absence or presence of 2.5 µM actin. Released chromatin was labelled with an anti-CitH3 Ab. (**B**) Comparison of NET formation by neutrophils stimulated with H, M, L IL-8, or HC supplemented with 100 pg/mL IL-8 with or without actin. (**C**) NET formation of H, M and L IL-8 serum. (**D**) cfDNA levels in burns IL-8 groups with or without actin. [*t*-test *p* value]: * < 0.05, ** < 0.01. (ns) not significant. Data are representative of n = 5.

To test whether serum samples were potentially degrading NETs, identical experiments were performed in the presence and absence of the specific DNase inhibitor, actin. Figure 4 illustrates that by inhibiting DNase I with actin, more extensive NET generation occurred (Figure 4A–C), with less liberation of cfDNA into the supernatants (Figure 4D).

### 2.4. IL-8 Is a Major Contributor to Burns Serum NET-Inducing Capacity

**Figure 5:**
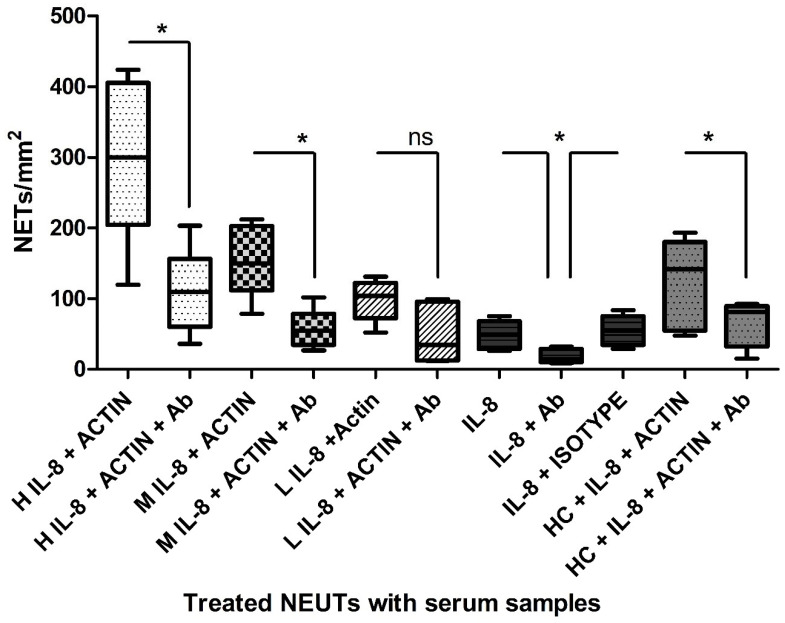
**The effect of anti-IL-8 antibodies on NET formation.** NET quantification induced by burns serum from high (H), medium (M) or low (L) IL-8 samples, recombinant IL-8, anti-IL-8 Ab isotype, or supplemented HC serum in the presence of 2.5 µM actin and with or without anti-IL-8 Ab. [*t*-test *p* value]: * < 0.05. (ns) not significant. Data are representative of n = 5.

To determine whether IL-8 is the predominant mediator of NETosis in burns serum, experiments were undertaken in the presence of an anti-IL-8 monoclonal antibody (mAb). Figure 5 illustrates that the addition of an anti-IL-8 mAb significantly reduced NET generation compared to an isotype control within H and M IL-8 serum samples (*p* < 0.05) and inhibited the action of recombinant IL-8 added to normal serum (*p* < 0.05).

### 2.5. IL-8 Levels Are Significantly Correlated with cfDNA Levels in Severe Thermal Injury

**Figure 6:**
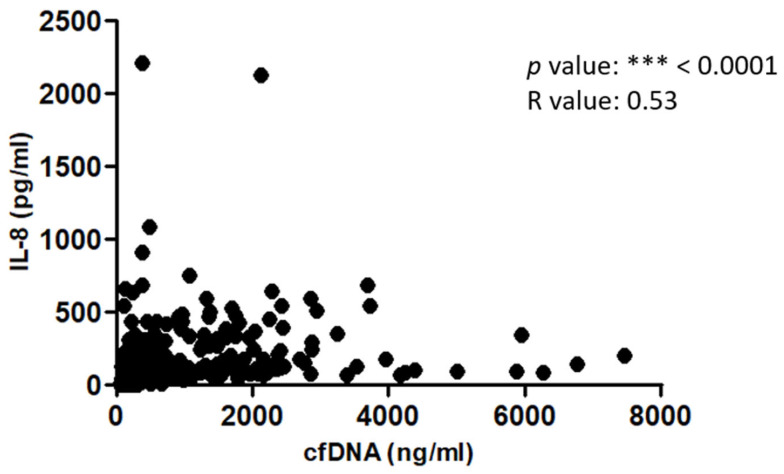
**The overall IL-8 correlation with cfDNA levels in thermal injuries.** Significant overall correlation between IL-8 and cfDNA levels in thermal injuries (n = 96). *p* value: *** < 0.0001.

We determined if there was a correlation between circulating IL-8 and cfDNA levels in burns. Table 1 shows a significant correlation between IL-8 and cfDNA levels in burns from admission day to month 3. The *p* value was <0.05 on days 1 and 3, and <0.001 on days 5, 7, 10, 12, 14, and 28 with *R* value ranging from 0.46 to 0.79. The overall correlation is also significant with a *p* value < 0.001, and an *R* value of 0.53 (Figure 6).

## 3. Discussion

NET formation was first described by Brinkmann et al. (2004) [6]. Neutrophils augment their antimicrobial capabilities via the release of NETs, which consist of extracellular chromatin adorned with histones and other granular proteins [37]. These NETs have been recognised as components of the innate immune response, potentially exerting a combination of therapeutic or pathogenic effects [6,37,38]. As NETs are important as mediators of DIC and MOF in burns patients with sepsis, they not only offer potential new prognostic biomarkers but point to a range of potential new therapies to either inhibit their formation or degradation [39]. Therefore, understanding the pathophysiological mechanisms by which NETs are generated and degraded is key for identifying and optimising new therapeutic targets.

In 2019, Abrams et al. demonstrated that high IL-8 levels in the serum from ICU patients were a major contributor to NET formation [34]. This has also been demonstrated in diffuse large B-cell lymphoma patients [35]. In this study, we show that IL-8 levels are significantly increased in patients with severe thermal injury on days 3 and 5 following injury compared to healthy controls. Comparison of levels between patients with and without sepsis also demonstrated increased levels at day 5 in patients who developed sepsis. Furthermore, IL-8 levels in patients who developed sepsis were significantly higher than healthy controls at the majority of time points from day 3 to day 14 following injury. Our findings confirm previous studies showing increased IL-8 levels following thermal injuries and its significant correlation with septic burns [40,41,42,43,44].

We next investigated the biological activity of serum containing low to high IL-8 levels on the capacity to induce ex vivo NET generation. Our initial results confirmed that incubating neutrophils with serum containing high concentrations of IL-8 induced NET formation in contrast to the negative control of healthy control serum. Supplementing healthy control serum with 100 pg/mL recombinant IL-8 as a positive control also confirmed the ability of IL-8 to induce ex vivo NET production. We then categorised the burns serum into three groups based on their IL-8 levels (low, medium and high) and investigated their capacity to induce NET generation. As expected, there was a dose–response effect correlating with the levels of serum IL-8, an observation that confirms the study by Abrams et al. in ICU patients [34].

Various studies have demonstrated the involvement of other pro-inflammatory mediators in the generation of NETs. For example, cytokines such as IL-1β and TNF-α have been shown to be involved in NET production in SIRS subjects [44,45,46]. Moreover, Itagaki et al. (2015) demonstrated significant NET generation by treating purified neutrophils with mitochondrial DNA [47]. In addition, Zhang et al. (2020) reported that IL-17 promotes NET production by recruiting neutrophils and triggering NETosis in pancreatic cancer patients [48]. Van Avondt et al. (2023) have shown that released iron from red blood cells (RBC) also contributes to NETosis [49]. Additionally, heme has been identified as a significant mediator that triggers neutrophil adhesion, which is dependent on nuclear factor-κappa B (NFκB) and ROS pathways [50]. Recently, Teng et al. (2024) emphasised the ability of interferon-gamma (IFNγ) to generate NETs and proposed its potential use in enhancing the activity of eliminating tumours in microsatellite stable colorectal cancer [51].

In the experiments we conducted on serum IL-8 dose–response effects on ex vivo NETosis, we also measured cfDNA in cell supernatants. Interestingly, these results confirmed a dose–response effect, with increased cfDNA correlating with high, medium and low levels of IL-8. However, given that DNase activity is also present within the serum, the data would also suggest that cfDNA is being released from NETs in the presence of serum, particularly as the PMA-positive control in the absence of serum induced the most NETs observed by microscopy but with a reduced amount of released cfDNA in the supernatant compared to serum samples. DNase activity is therefore a potential confounding variable when using serum samples in these experiments. We therefore tested this hypothesis by performing identical experiments in the presence and absence of a specific DNase inhibitor actin. These experiments confirmed that the inhibition of DNase caused a significant increase in NET formation as observed by microscopy. However, this also reduced the release of cfDNA in the supernatants and demonstrates that ex vivo experiments on NETs using serum as an inducer ideally need to take this into account and may underestimate the true magnitude of NET formation measured.

Major tissue damage following severe burns results in the excessive release of circulating actin from tissues, which directly inhibits DNase I [21,52,53]. Dinsdale et al. (2020) previously demonstrated that actin reduces DNase I activity and that the actin scavenging system normally protects DNase I activity, which is important for NET homeostasis. [20]. In line with these observations, we found that in vitro inhibition of DNase I activity by actin resulted in increased NET formation with less degradation into cfDNA in the supernatants. A limitation of this study is that we were unable to study any potential relationship between IL-8 and actin levels in burns samples as the presence of actin was only qualitatively analysed by Western blotting in our previous study (Dinsdale et al., 2020) [20].

To determine whether IL-8 is the predominant mediator of NETosis in burns serum, experiments were undertaken in the presence of an anti-IL-8 monoclonal antibody. The addition of an anti-IL-8 mAb not only significantly reduced NET generation by high and medium IL-8 serum samples (*p* < 0.05) but also inhibited the action of recombinant IL-8 added to normal serum (*p* < 0.05). Given the magnitude of the inhibition observed, it seems likely that IL-8 is a major contributor to NET formation in thermal injury both in vivo and within ex vivo assays but also suggests that other mediators are likely to be involved.

Our results suggest that increased IL-8 levels may contribute to excessive NETosis that subsequently results in cfDNA elevation levels in thermal patients due to NET degradation by DNase. Previously, we have demonstrated that cfDNA levels are also elevated post-thermal injury [54]. Therefore, we evaluated the relationship between IL-8 and cfDNA levels in burns. Our data demonstrate a significant correlation between IL-8 and cfDNA levels in burns from admission day to month 3. The overall correlation was also significant, suggesting that high IL-8 levels significantly contribute to increased NET-derived circulating cfDNA levels, which are a pro-inflammatory damage-associated molecular pattern (DAMP) biomarker for DIC and MOF [16,18,55].

In conclusion, this study demonstrates the importance of serum IL-8 in inducing NET formation following thermal injury. Levels of circulating IL-8 were shown not only to be increased in patients with burn injury, but also to be increased in patients that developed sepsis. Furthermore, an ex vivo dose–response effect was confirmed that could be inhibited by anti-IL-8 antibodies. Inhibition of DNase activity in serum by actin not only confirms the importance of this enzyme in the regulation of NETs but also demonstrates its potential as a confounding variable in serum-based neutrophil stimulation assays.

## 4. Materials and Methods

### 4.1. Study Design

The Scientific Investigation of the Biological Pathways Following Thermal Injury-2 (SIFTI-2) is an ongoing observational cohort study at Queen Elizabeth University Hospital Birmingham enrolling patients with moderate-to-severe burn injuries: ≥15% and ≥20% of the total body surface area (TBSA) in adults and children, respectively [56]. Patients are admitted and recruited through the major trauma and burns centres at the Queen Elizabeth Hospital Birmingham. Daily blood samples are collected from patients from hospital admission to day 14 post-burn, as well as at day 28, and months 3, 6, 12 and 24 post-injury. Clinical data are collected and held in anonymous databases that contain clinical assessments, injury severity and key biomarkers. Sepsis was diagnosed according to classical ABA criteria [57].

This study was approved by the research ethics committee (REC reference:16/WM/0217) [56].

### 4.2. Patient Cohort

A total of 96 burn patients were included in the study. The median age of patients was 49 years (range 16–84 years), and the median burn size was 32% total body surface area (TBSA) (range 15–85%). The incidence of sepsis was 51.2% and mortality was 20.8%. Septic status was not determined for 12 patients due to early mortality from non-septic causes or study withdrawal. Detailed patient demographics are displayed in Table 2.

### 4.3. Blood Sampling and Preparation of Serum and Plasma

Blood samples were collected from 96 thermally injured patients (TBSA ≥ 15%) at days 1–14, day 28, and months 3, 6 and 12 post-injury. Blood samples were collected into vacutainers containing sodium citrate or z-serum clotting activator for the preparation of platelet-free plasma and serum, respectively. To generate serum, vacutainers were left at room temperature for 30 min, centrifuged at 1620× *g* for 10 min, and the serum was aliquoted and stored at −80 °C. Platelet-free plasma was isolated from sodium citrate tubes as described by Hampson et al. (2017) [54].

### 4.4. Measurement of IL-8 Levels

IL-8 levels in healthy controls (HC; N = 10) and burns patients were measured in serum samples. Samples were diluted 1:4 with 1% BSA (Bovine Serum Albumin) (Sigma-Aldrich, Poole, UK) in PBS (Phosphate-Buffered Saline) (Sigma-Aldrich, Poole, UK) and analysed using a multiplex assay according to manufacturer’s instructions (Bio-Rad, Hertfordshire, UK).

### 4.5. Ex Vivo NET Generation

Blood samples were collected from healthy volunteers into EDTA tubes through venepuncture of the antecubital vein. A complete blood count (CBC) test was performed for each sample using a full-blood cell counter (XN1000, Sysmex UK, Milton Keynes). Blood was then transferred into 50 mL Falcon tubes containing 2% dextran T-500 in normal saline to sediment erythrocytes for 40 min. The neutrophils were extracted using a discontinuous density gradient of Percoll (Sigma-Aldrich, Poole, UK). The neutrophils were suspended in RPMI 1640 medium (Sigma-Aldrich, Poole, UK) containing 10% heat-inactivated foetal bovine serum, 2 mM glutamine, 100 U/mL penicillin, and 100 μg/mL streptomycin (Sigma-Aldrich, Poole, UK) and counted within the XN1000. The final concentration of isolated neutrophils was made at 20 × 10^6^/mL.

NET generation was performed by stimulating neutrophils with burn serum samples categorised as having either low (<150 pg/mL), medium (150–500 pg/mL), or high (>500 pg/mL) levels of IL-8 based on the description d by Abrams et al. (2019) [34]. In brief, patient or healthy control serum (90 µL) was incubated with 10 µL purified neutrophils (20 × 10^6^/mL) from healthy individuals for 4 h at 37 °C and 5% CO_2_ in glass chamber slides (BD Biosciences, UK). Untreated and treated neutrophils stimulated with 25 nM phorbol myristate acetate (PMA) in RPMI 1640 Medium (ThermoFisher, Cheshire, UK) supplemented with 2 mM glutamine, 100 U/mL penicillin, and 100 μg/mL streptomycin were performed as negative and positive controls, respectively. Recombinant human IL-8 (Fine-Test Biotech, Boulder, US) (100 pg/mL) was also used to induce NETosis in RPMI 1640 as a positive control. Post-incubation, cells were fixed with 2% paraformaldehyde (Sigma-Aldrich, Poole, UK) for 30 min. After fixation, supernatants were collected into 0.5 mL Eppendorf tubes. Cells were washed with sterile PBS and stained for 5 min with 5 µM SYTOX green (ThermoFisher, Cheshire, UK). After washing, cells were incubated with 1% BSA in PBS for one hour. Extracellular NETs were incubated with 1 µg/mL anti-citrullinated histone antibody (anti-citH3 Ab) (Abcam, Cambridge, UK) or an isotype control (Abcam, Cambridge, UK) overnight at 4 °C. Cells were then washed with 1% BSA in PBS and stained with 0.1 µg/mL Donkey anti-Rabbit IgG secondary antibody conjugated to Alexa Fluor™ 488 (ThermoFisher, Cheshire, UK) for one hour. Lastly, cells were washed with 1% BSA in PBS and visualised by immunofluorescence microscopy (×20 magnification). In some samples, 1 µg/mL of human IL-8 monoclonal antibody (R&D Systems, MAB208-100, Abingdon, UK) or a mouse IgG1 isotype control (R&D systems, Abingdon, UK) was incubated for 15 min in isolated neutrophils at 37 °C and 5% CO_2_ before treating cells with serum or recombinant IL-8 to inhibit the production of NETs by IL-8To inhibit potential degradation of generated NETs by DNase I, some serum samples were also treated with 2.5 µM Actin (Sigma-Aldrich, Poole, UK) for 30 min prior to incubation. Generated NETs were semi-quantified by fluorescence microscopy using ImageJ software.

Cell-free DNA (cfDNA) levels in supernatants were measured according to the method by Goldshteub et al. (2009) [58]. The supernatants of untreated and treated samples, after fixation, were collected into 0.5 mL Eppendorf tubes and centrifuged at 2200× *g* for 2 min. For more accuracy, healthy and burn serum samples were incubated alone without neutrophils in different wells with similar conditions as controls for cfDNA levels of treated neutrophil samples. After centrifugation, 50 µL of cell-free supernatants was transferred to wells of a 96-well flat-bottomed black plate (Corning, Flintshire, UK) and stained for 10 min at room temperature with 0.5 mM SYTOX green. Lambda DNA (ThermoFisher, Cheshire, UK) was included to generate a λ-DNA standard curve ranging from 1000 ng/mL to 0 ng/mL. Fluorescence was measured using a BioTek Synergy 2 fluorometric plate reader (NorthStar Scientific Ltd., Sandy, UK) with excitation and emission set at 485 and 528 nm, respectively.

### 4.6. Statistics

Statistical analysis was performed using GraphPad Prism software. Data distribution was determined using a Kolmogorov–Smirnov test. IL-8 concentrations at each timepoint were compared to HCs (n = 10) values using a Kruskal–Wallis test. IL-8 levels of septic and non-septic burns patients were compared via Two Way ANOVA test. Generated NETs and chromatin were compared by One Way ANOVA test. The comparison between treated groups in NETs or cfDNA generation in the presence or absence of actin and anti-IL-8 Ab was made via a Student’s *t*-test. The relationship continuous values between IL-8 and cfDNA were measured by Spearman correlation test. Generated NETs were semi-quantified by microscopy using ImageJ software (version 1.54f). A statistically significant result was defined with a *p* value < 0.05.

## Figures and Tables

**Table 1:** Correlation between IL-8 and cfDNA levels in thermal injuries (n = 96).

Days	Number of Burn Patients	Correlation (Y/N)	*p* Value	R Value
DAY 1	78	Y *	0.0259	0.2523
DAY 3	69	Y *	0.0425	0.2449
DAY 5	69	Y ***	<0.0001	0.4578
DAY 7	66	Y ***	<0.0001	0.5096
DAY 10	60	Y ***	<0.0001	0.6917
DAY 12	59	Y ***	<0.0001	0.7687
DAY 14	57	Y ***	<0.0001	0.6744
DAY 28	38	Y ***	<0.0001	0.7902
Month 3	10	Y **	0.0036	0.8207
Overall	506	Y ***	<0.0001	0.53

*p* value *** < 0.001, ** < 0.01, * < 0.05.

**Table 2:** Patient demographics.

Characteristic	Burns Patients (n = 96)
Age, years (range)	49 (16–84)
Gender, (M:F)	74:22
% TBSA (range)	32 (15–85)
% FT TBSA (range)	11 (0–80)
Inhalation injury (Y:N)	39:57
Mechanism of injury	
Flash, n (%)	7 (7.29)
Flame, n (%)	79 (82.29)
Flame and flash, n (%)	5 (5.20)
Electrical, n (%)	1 (1.04)
Scald, n (%)	4 (4.17)
ABSI (range)	8 (2–14)
Baux (range)	80 (34–143)
rBaux (range)	89 (39–160)
SOFA	6 (0–17)
Sepsis Y:N (%)	43:41 (51.2)
Mortality Y:N (%)	20:76 (20.83)

Abbreviations: TBSA (total body surface area), FT (Full-thickness), ABSI (A Body Shape Index), Baux (a burn mortality prediction system), rBaux (revised Baux indication), SOFA (Sequential Organ Failure Assessment).

## Data Availability

Data will be mad e available by the authors upon reasonable request.
